# MicroRNA-9 induces defective trafficking of Nav1.1 and Nav1.2 by targeting Navβ2 protein coding region in rat with chronic brain hypoperfusion

**DOI:** 10.1186/s13024-015-0032-9

**Published:** 2015-08-11

**Authors:** Li-Hua Sun, Mei-Ling Yan, Xue-Ling Hu, Li-Wei Peng, Hui Che, Ya-Nan Bao, Fei Guo, Tong Liu, Xin Chen, Rong Zhang, Tao Ban, Ning Wang, Huai-Lei Liu, Xu Hou, Jing Ai

**Affiliations:** Department of Pharmacology, Harbin Medical University, No.157 Baojian Road, Nangang District,Harbin, Heilongjiang Province, 15008 China; Department of Neurosurgery, The First Affiliated Hospital of Harbin Medical University, No.157 Baojian Road, Nangang District, Harbin, Heilongjiang Province 150081 China

**Keywords:** microRNA-9, Chronic brain hypoperfusion, Sodium channel

## Abstract

**Background:**

Previous studies have demonstrated that the trafficking defects of Nav1.1/Nav1.2 are involved in the dementia pathophysiology. However, the detailed mechanisms are not fully understood. Moreover, whether the impaired miRNAs regulation linked to dementia is a key player in sodium channel trafficking disturbance remains unclear. The cognitive impairment induced by chronic cerebral ischemia through chronic brain hypoperfusion (CBH) is likely reason to precede dementia. Therefore, our goal in the present study was to examine the role of microRNA-9 (miR-9) in regulating Nav1.1/Nav1.2 trafficking under CBH generated by bilateral common carotid artery occlusion (2VO).

**Results:**

The impairment of Nav1.1/Nav1.2 trafficking and decreased expression of Navβ2 were found in the hippocampi and cortices of rats following CBH generated by bilateral 2VO. MiR-9 was increased in both the hippocampi and cortices of rats following CBH by qRT-PCR. Intriguingly, miR-9 suppressed, while AMO-miR-9 enhanced, the trafficking of Nav1.1/Nav1.2 from cytoplasm to cell membrane. Further study showed that overexpression of miR-9 inhibited the Navβ2 expression by targeting on its coding sequence (CDS) domain by dual luciferase assay. However, binding-site mutation or miR-masks failed to influence Navβ2 expression as well as Nav1.1/Nav1.2 trafficking process, indicating that Navβ2 is a potential target for miR-9. Lentivirus-mediated miR-9 overexpression also inhibited Navβ2 expression and elicited translocation deficits to cell membrane of Nav1.1/Nav1.2 in rats, whereas injection of lentivirus-mediated miR-9 knockdown could reverse the impaired trafficking of Nav1.1/Nav1.2 triggered by 2VO.

**Conclusions:**

We conclude that miR-9 may play a key role in regulating the process of Nav1.1/Nav1.2 trafficking via targeting on Navβ2 protein in 2VO rats at post-transcriptional level, and inhibition of miR-9 may be a potentially valuable approach to prevent Nav1.1/Nav1.2 trafficking disturbance induced by CBH.

## Background

Since voltage-gated sodium channel (VGSC) is necessary in the initiation and propagation of action potentials in neurons, it is a valuable therapeutic target for neurological disorders, such as epilepsy and chronic neuropathic pain [1–3]. Recent studies have now expanded the role of sodium channels in multi-neurological diseases including autism, migraine and multiple sclerosis [2, 4, 5]. A prospective study reported that Alzheimer's disease (AD) have an increased risk of developing seizures and epilepsy [6]. A recent study reported that over activity of hippocampus might contribute to AD-related cognitive decline [7] and antiepileptic drug was demonstrated to reverse cognitive deficits and diminished the anxiety phenotypes in AD mice [8]. And electrical imbalance may contribute to cognitive deficits in AD and serve as a target for clinical intervention [9, 10]. These studies together suggest that dysfunction of VGSC may share the common phenotype between AD and epilepsy.

Voltage-gated Na^+^ channels (VGSCs) are macromolecular protein complexes, which are composed of α-subunits (Nav1.1–Nav1.9) and β-subunits (Navβ1, Navβ1B, Navβ2, Navβ3 and Navβ4), in which α-subunits are necessary for forming a functional ion-selective channel and β-subunits affect ion channel gating and trafficking to regulate the voltage-dependency and density of VGSC on the cell membrane. It has been reported that mutation of Nav1.1, a remarkable feature of Dravet syndrome, could induce higher seizure activity and cognitive dysfunction [11]. Interestingly, Nav1.1 retained inside the cells while its expression reduces markedly in *BACE1*-transgenic mice, accompanied with disturbed Navβ2 [12]. And restoring Nav1.1 level could reduce memory deficits in human amyloid precursor protein (*hAPP)* transgenic mice [13]. Furthermore, elevated cell surface Nav1.2 expression contributes to the epileptic behaviors in *BACE1*-null mice [14]. These studies indicated that the abnormal Nav1.1/Nav1.2 trafficking may be involved in dementia. Navβ2 plays a key role in the trafficking of α-subunits from cytoplasm to cell membrane [15–17], and keeping the steady-state stabilization of VGSC complexes at the plasma membrane [18]. Interestingly, Navβ2 is also one substrate for beta-secretase (*BACE1*) in *BACE1*-deficient or over-expressing mice [19], and the increased Navβ2 cleavage contributes to aberrant neuronal activity and cognitive deficits in amyloid precursor protein (*APP*) mice [20]. However, the mechanism remains largely unclear.

Chronic brain hypoperfusion (CBH)-mediated chronic cerebral ischemia and consequent cognitive impairment [21, 22] is most likely to precede dementia [23, 24]. It has been reported that CBH not only induces the accumulation of beta-amyloid (Aβ) [21, 24] and cell death [25], but also reduces dendritic arborizations as well as synaptic contacts [26]. And acute cerebral ischemia by the occlusion of right middle cerebral artery downregulates the total Nav1.1 protein expression from 6 h to 2 days, which was further increased from 3 to 7 days [27]. However, whether and how CBH influences the expression or trafficking of Nav1.1/Nav1.2 has not been reported.

MicroRNAs are small non-coding RNA, which regulate protein synthesis. MicroRNA-9 (miR-9), enriched in central nerve system (CNS) [28], contributes likely to multi-pathological processes including the neurogenesis [29], proliferation [30], migration and differentiation of neural progenitor cells [31], drug adaption [32], adult brain plasticity [33], neural cell fate [34], the migration and proliferation of glioma cells [35], axon extension and branching [36], spinal motor neuron development [37] under physiological status. Importantly, miR-9 expression has been downregulated in the brain of patients with Huntington's disease [38] and upregulated in the patients with Alzheimer's disease [39], suggesting that abnormal expression of miR-9 may be involved at least partially in the processes of neurodegenerative diseases. Therefore, whether and how miR-9 participates in the abnormal expression or trafficking of Nav1.1/Nav1.2 induced by CBH is worth to be explored.

In this study, our data provide strong evidence that miR-9 regulates Nav1.1/Nav1.2 trafficking by post-transcriptional regulating SCN2B gene under CBH status.

## Results

### CBH-mediated Nav1.1/Nav1.2 trafficking defect in the hippocampi and cortices

**Fig. 1:**
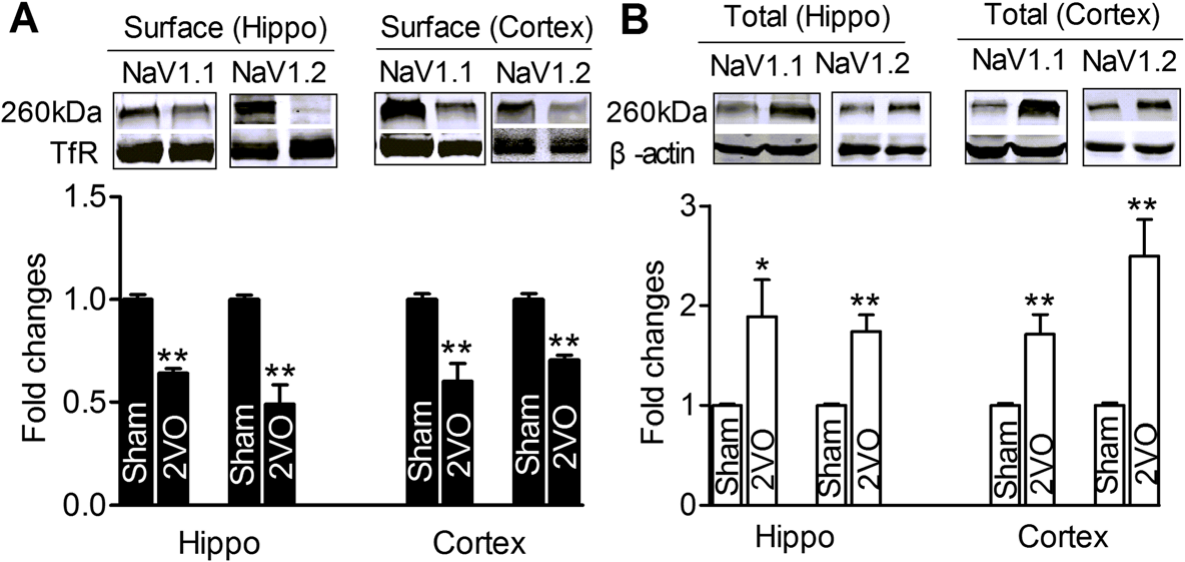
Nav1.1 and Nav1.2 trafficking were disturbed in hippocampi and cortices after chronic brain hypoperfusion (CBH). **a**, western-blot analysis of the surface protein levels of Nav1.1 and Nav1.2 in sham and 2VO rats, upper: representative immunoblots of Nav1.1 and Nav1.2; lower: the quantitative analysis data of the immunoblots. The optical density was evaluated for each band and values for 2VO rat tissue were normalized to sham group after correction for protein loading with TfR,**P < 0.01 *vs* sham, mean ± s.e.m, *n* = 6. **b**, western-blot analysis of the total protein levels of Nav1.1 and Nav1.2 in sham and 2VO rats, upper: representative immunoblots of Nav1.1, Nav1.2; lower: the quantitative analysis data of the immunoblots. The optical density was evaluated for each band and values for 2VO rat tissue were normalized to sham group after correction for protein loading with β-actin. **P < 0.01 *vs* sham, mean ± s.e.m, *n* = 6

Previous studies have reported that Nav1.1/Nav1.2 trafficking was changed after dementia [19, 20, 40]. Since the functional protein of Nav1.1/Nav1.2 located in the membrane, we first evaluated the surface expression of Nav1.1/Nav1.2 and found that the surface expression of Nav1.1 and Nav1.2 was reduced both in the hippocampi and cortices of 2VO rats (Fig. 1a, *P* < 0.01 *vs* sham). In order to explore whether the decreased surface protein expression of Nav1.1/Nav1.2 was due to the reduction of total Nav1.1/Nav1.2 or the impairment of trafficking, the total protein levels of Nav1.1/Nav1.2 were evaluated. The data showed that the total protein levels of Nav1.1 and Nav1.2 were significantly increased in the hippocampi and cortices of 2VO rats (Fig. 1b, *P* < 0.01 *vs* sham) rather than reduced. These results suggested that the decreased expression of Nav1.1/Nav1.2 on the cell membrane were due presumably to the impaired trafficking of Nav1.1 and Nav1.2 protein from cytoplasm to cell membrane in 2VO rats rather than the changes of their total protein levels.

### CBH-mediated Navβ2 downregulation in hippocampi and cortices

**Fig. 2:**
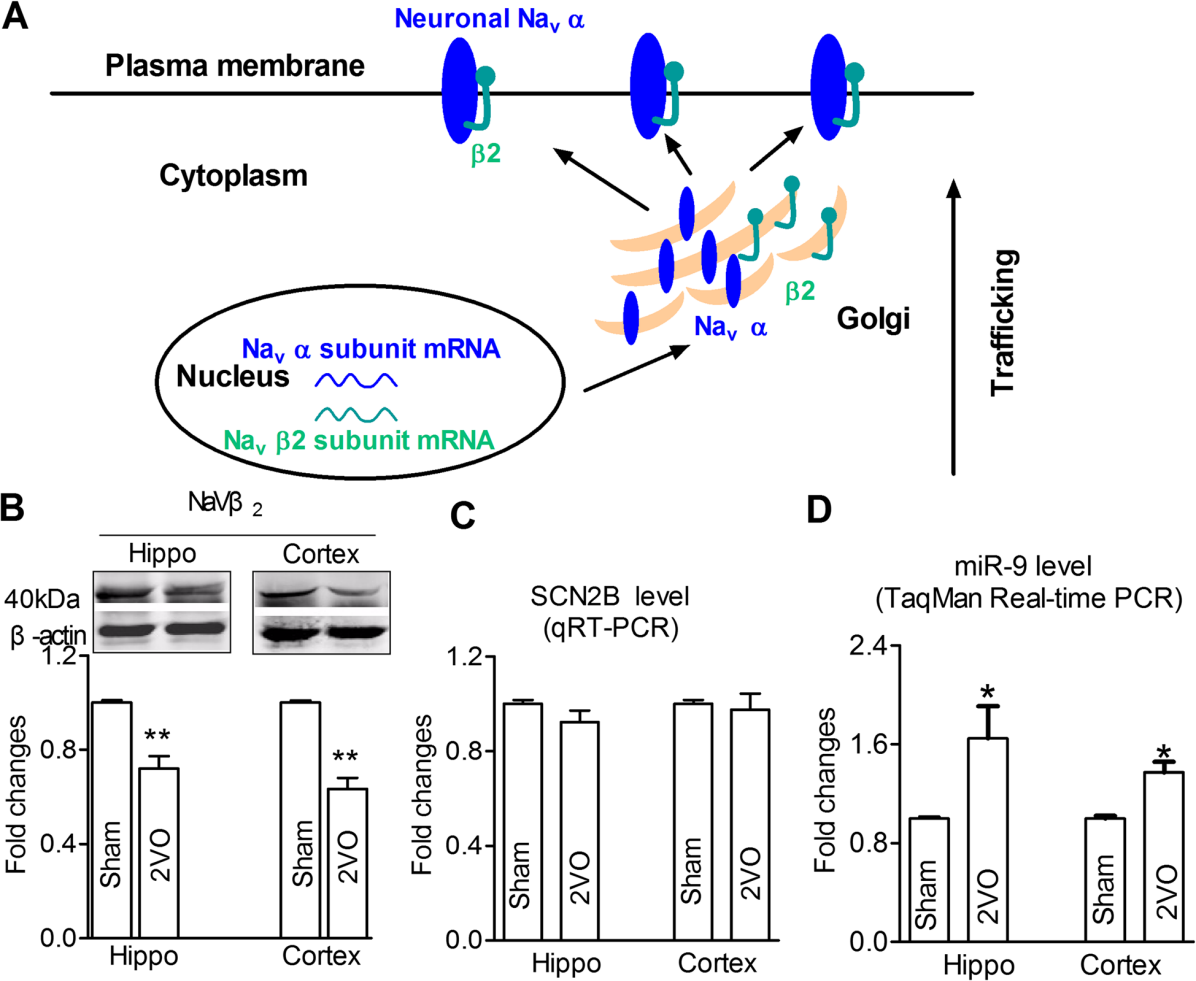
Navβ2 was decreased in hippocampi and cortices of rats after CBH. **a**, a model for Nav α-subunit trafficking after covalently binding to Navβ2 subunit. **b**, western-blot analysis of Navβ2 in sham and 2VO rats, upper: representative immunoblots of Navβ2; lower: the quantitative analysis data of the immunoblots. The optical density was evaluated for each band and values for 2VO rat tissue were normalized to sham group after correction for protein loading with β-actin. **P < 0.01 *vs* sham, mean ± s.e.m, *n* = 6. **c**, The relative quantification of Navβ2 mRNA (SCN2B) levels was detected by real-time PCR with normalized to β-actin, followed by further normalization to the values from sham brain tissues. *P* > 0.05 *vs* Sham, mean ± s.e.m, *n* = 3. **d**, miR-9 level detected by TaqMan real-time PCR in hippocampi and cortices from sham and 2VO rats after normalization to U6 levels. *P < 0.05 *vs* sham, mean ± s.e.m, *n* = 3

Since Navβ2 is considered as an important regulator for trafficking Nav1.1/Nav1.2 upon reports from others [16, 17] and our current observation (Fig. 2a), we then investigate the expression of Navβ2 in either hippocampus or cortex of 2VO rats. The protein expression of Navβ2 was significantly decreased in both brain locations compared with that in sham rats (Fig. 2b, *P* < 0.01). However, their mRNA expression of Navβ2 (SCN2B) was not changed consistently with the protein expression of Navβ2 (Fig. 2c), suggesting that this may be ascribed to the post-transcriptional regulation.

### MiR-9-mediated post-transcriptional regulation of Navβ2 expression

MicroRNAs have long been known to control post-transcriptional gene regulation and are essential for neuronal function [41]. MiR-9 is enriched in CNS, and increases in the brain of the patients with Alzheimer's disease (AD) [39].To test potential involvement of miR-9 in CBH-mediated 2VO rats, we also evaluated the expression of miR-9 in hippocampi and cortices of 2VO rats. Surprisingly, the expression levels in both brain regions were significantly increased compared with those in sham control rats quantified using qRT-PCR (Fig. 2d, *P* < 0.05).

**Fig. 3:**
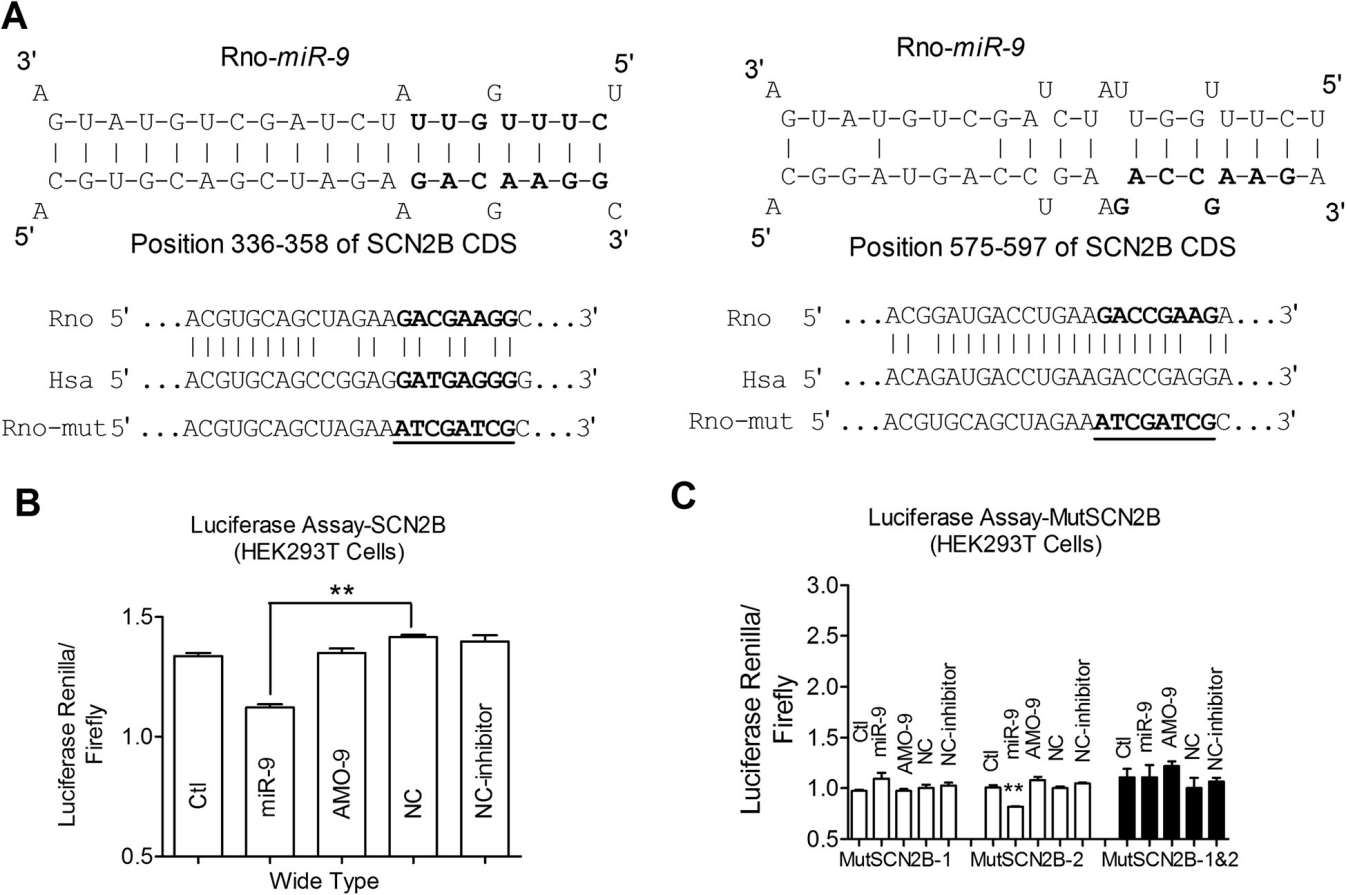
miR-9 regulates SCN2B evaluated by luciferase array. **a**, Complementarity between miR-9 seed-matched sequence and the region coding for Navβ2 predicted by a computational and bioinformatics-based approach using RNA22/RNAhybrid. Two binding sites were found at the position of 336–358 of SCN2B CDS and the position of 575–597 of SCN2B CDS. The mutation made to genes are underlined. **b**, Luciferase reporter gene assay for interactions between miR-9 and its binding sites in the CDS region of the Navβ2 mRNA in HEK293T cells. Cells were transfected with luciferase-target motif chimeric vector alone, miR-9, AMO-9, or scramble negative control (NC) using lipofectamine 2000. **P < 0.01 *vs* NC, mean ± s.e.m. NC-inhibitors means scramble negative inhibitor control, *n* = 3. **c**, Luciferase reporter gene assay for interactions between miR-9 and mutation of binding site in the CDS region of SCN2B in HEK293T cells, **P < 0.01 *vs* NC, mean ± s.e.m, *n* = 3

Since the protein level of Navβ2 was reduced in both hippocampi and cortices in 2VO rats, which is discrepancy with the unchanged SCN2B expression, the alternation of miR-9 is highly expected in this process. By searching the database of microRNA targets (http://www.targetscan.org/), we found that SCN2B has a poorly conservative ‘seed’ sequence of miR-9 in its 3’UTR and the length of 3’UTR of SCB2B in rat was very short based on the database of the University of California Santa Cruz (UCSC) and the National Center for Biotechnology Information (NCBI) Genome Browsers. Since recent studies have reported that microRNAs regulate protein expression by binding to the coding regions of target protein [41–43], we then searched RNA22/RNA hybrid (http://bibiserv.techfak.uni-bielefeld.de/rnahybrid/) and found that the CDS domain of SCN2B is likely to serve as potential targets for miR-9. We next identified whether there are binding sites for miR-9 on the CDS domain of SCN2B gene at the position of 336–358 and 575–597 with highly conservative regions (Fig. 3a). We subsequently screened the predicted SCN2B binding sites using luciferase assays (see Methods) and then selected constructs including position 336–358 and position 575–597 of SCN2B CDS domains that substantially suppressed luciferase expression in the presence of the matching microRNAs (Fig. 3b). MiR-9 inhibited the SCN2B-CDS luciferase activity compared with scrambled negative control (Fig. 3b, *P* < 0.01), however, scrambled negative control and inhibitor of miR-9 had no effect on luciferase activity compared with control group (Fig. 3b, *P* > 0.05), and the difference of luciferase activity between inhibitor of miR-9 and scramble negative inhibitor control was also not detected (Fig. 3b, *P* > 0.05). For each of the two binding sites, we generated and tested several variants by introducing silent mutations designed to disrupt the putative base pairings between the microRNAs and the corresponding predicted targets. Silent mutations introduced into the predicted targets at the position of 336–358 of SCN2B CDS disrupted the ability of miR-9 to repress the translation of the SCN2B-CDS (Fig. 3c, *P* < 0.01). However, simultaneous introduction of silent mutations at the position of 575–597 of SCN2B CDS did not abolish the downregulation of SCN2B by miR-9 (Fig. 3c, *P* > 0.05). Silent mutations introduced into both two predicted targets at the positions of 336–358 and 575–597 of SCN2B CDS, MutSCN2B-1 & 2 disrupted the ability of miR-9 to repress the translation of the SCN2B-CDS, strongly suggesting that miR-9 uniquely targets at the position of 336–358, rather than 575–597, of SCN2B CDS.

**Fig. 4:**
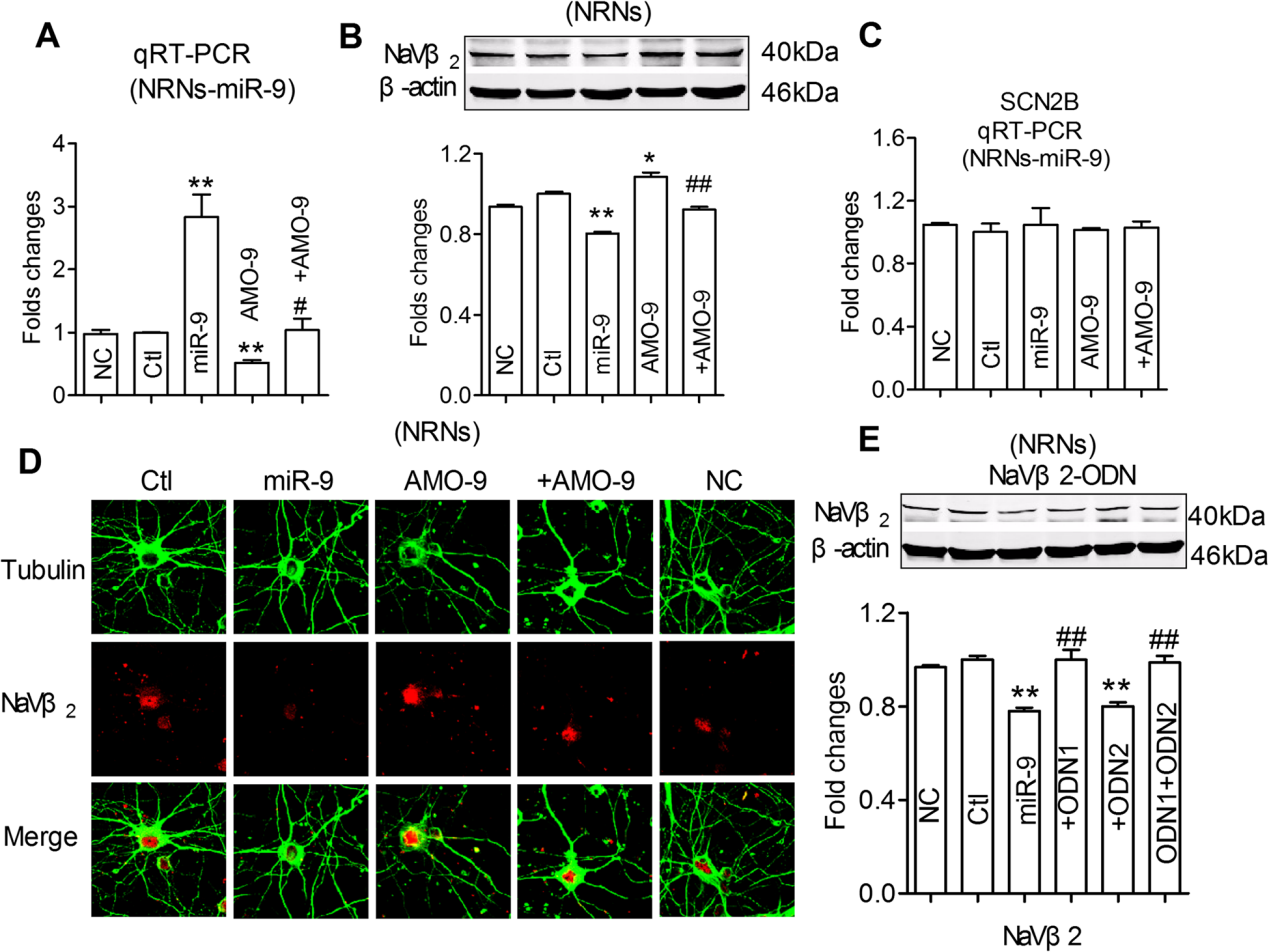
MiR-9 downregulates the expression of Navβ2 proteins. **a**, Verification of uptake of miR-9 by NRNs after transfection, *P < 0.05 *vs* NC, ^#^ *P* < 0.05,  mean ± s.e.m, *n* = 3 independent RNA samples for each group. **b**, Effects of miR-9 on protein levels of endogenous Navβ2 in primary cultured neonatal rat neurons (NRNs), using western blot analysis. Cells were transfected with miR-9, AMO-9, miR-9 + AMO-9, or NC. mean ± s.e.m from 3 batches of cells for each group. *P < 0.05, **P < 0.01 *vs* NC; ^##^ *P* < 0.01 *vs* miR-9. **c**, Effects of miR-9 on SCN2B in primary cultured neonatal rat neurons (NRNs) using qRT-PCR analysis. Cells were transfected with miR-9, AMO-9, miR-9 + AMO-9, or NC. mean ± s.e.m from 3 batches of cells for each group. **d**, Representative confocal microscope images showing primary culture hippocampus neuron stained for Tubulin (green, upper), Navβ2 (red, middle). A merged image depicting double positivity (yellow) is shown on the bottom after transfection with miR-9 mimics or/and AMO-miR-9, negative control, *n* = 3. **e**, Repression of Navβ2 by miR-9 using the miRNA-masking antisense oligodeoxynucleotides (ODNs) techniques in NRNs determined by Western blot analysis, *n* = 3 batches of cells for each group, mean ± s.e.m, **P < 0.01 *vs* NC; ^##^ *P* < 0.01 *vs* miR-9, ODN1 (oligodeoxynucleotides, which masks the binding sites of miR-9, located in the position 336–358 of SCN2B CDS region); ODN-2 (oligodeoxynucleotides, which masks the binding sites of miR-9, located in the position of 575–597 of SCN2B CDS region)

To observe the influence of miR-9 on the protein translation of SCN2B, we analyzed the protein levels of Navβ2 in primary cultured neonatal rat neurons (NRNs) co-transfected with miR-9 mimics. The successful transfection of miR-9 was identified (Fig. 4a) by qRT-PCR and Navβ2 protein levels were significantly decreased in the presence of miR-9 (Fig. 4b). AMO-9 rescued the downregulation of Navβ2 elicited by overexpression of miR-9 and scrambled negative control of microRNA failed to affect the protein levels, suggesting that miR-9 predominantly suppresses SCN2B translation (Fig. 4b,d). However, the mRNA level of Navβ2 was not changed in the presence of miR-9 (Fig. 4c), which may ascribed to transcriptional regulation. To verify the transcriptional mechanism of miR-9 on Navβ2, the target protector technique was applied and the results showed that the SCN2B target protector (oligodeoxynucleotides (ODNs)-miR) of miR-9 (the position of 336–358 of SCN2B CDS) attenuated the reduction in Navβ2 levels induced by miR-9 (Fig. 4e, *P* < 0.01), implying that the SCN2B shows a great potential as the target for miR-9.

### MiR-9-induced increase of total Nav1.1 /Nav1.2 protein in vitro

**Fig. 5:**
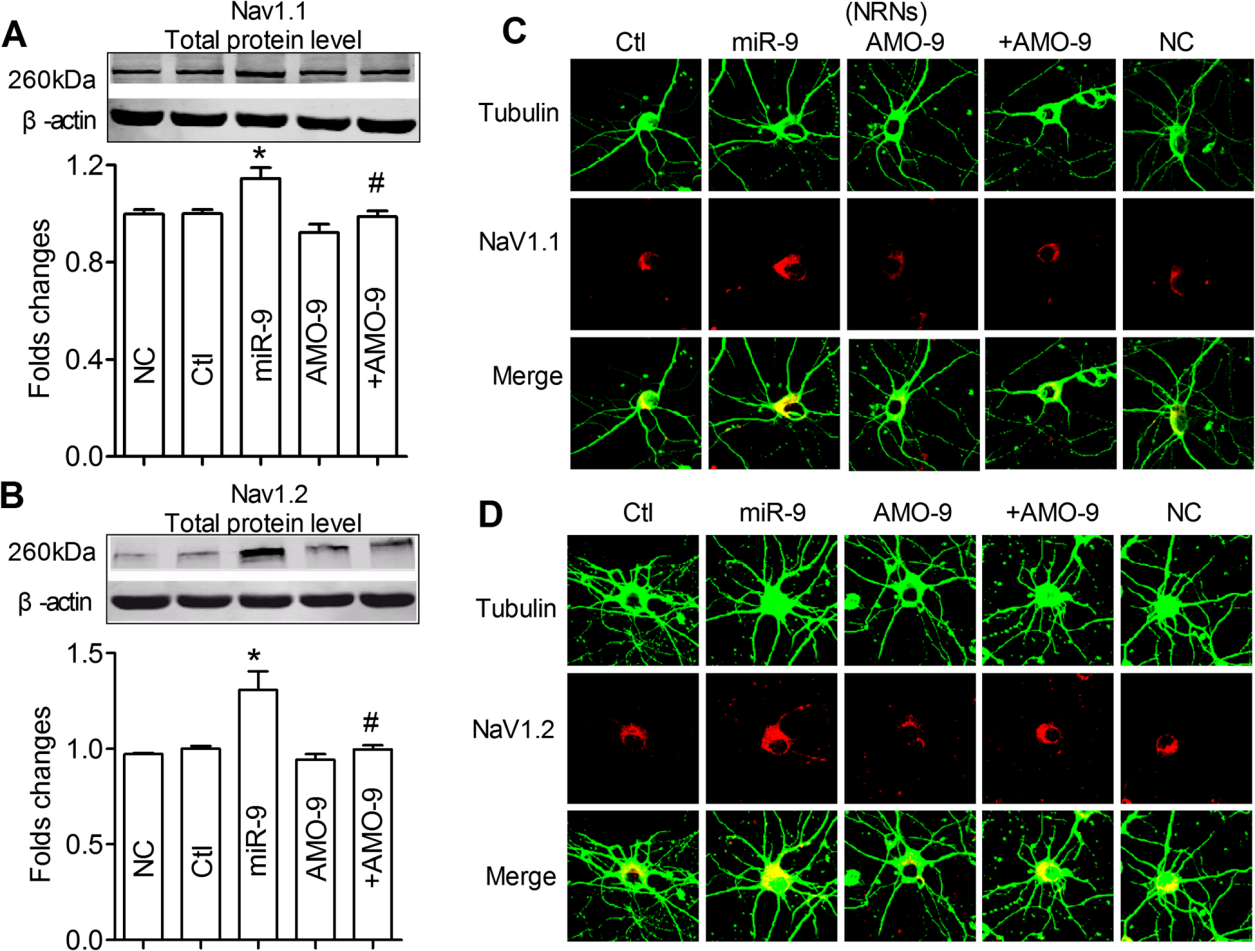
MiR-9 induced the increased of total Nav1.1 and Nav1.2 in primary cultured neonatal rat neurons (NRNs). **a**,**b** Effects of miR-9 on total protein levels of endogenous Nav1.1 (**a**), Nav1.2 (**b**), in NRNs, using western blot analysis and . Cells were transfected with miR-9, AMO-9, miR-9 + AMO-9, or NC. mean ± s.e.m from 3 batches of cells for each group. *P < 0.05 *vs* NC; ^#^ *P* < 0.05 *vs* miR-9. **c**-**d**,Representative confocal microscope images showing primary culture hippocampus neuron stained for Tubulin (green, upper), Nav1.1 (C)/Nav1.2 (D) (red, middle). A merged image depicting double positivity (yellow) is shown on the bottom after transfection with miR-9 mimics or/and AMO-miR-9, negative control

To test the trafficking effect of miR-9 on Nav1.1/Nav1.2, the protein expressions of Nav1.1 and Nav1.2 after pretreatment of NRNs with miR-9 were detected by both Western blot and immunofluorescence techniques. The results showed that miR-9 significantly increased the expression of of Nav1.1 (Fig. 5a, *P* < 0.05) and Nav1.2 (Fig. 5b, *P* < 0.05) total protein. And they were prevented in the presence of AMO-9 (Fig. 5a,b). The results were further observed by immunofluorescent analysis (Fig. 5c,d).

### MiR-9-induced disturbances of Nav1.1/Nav1.2 trafficking *in vivo*

**Fig. 6:**
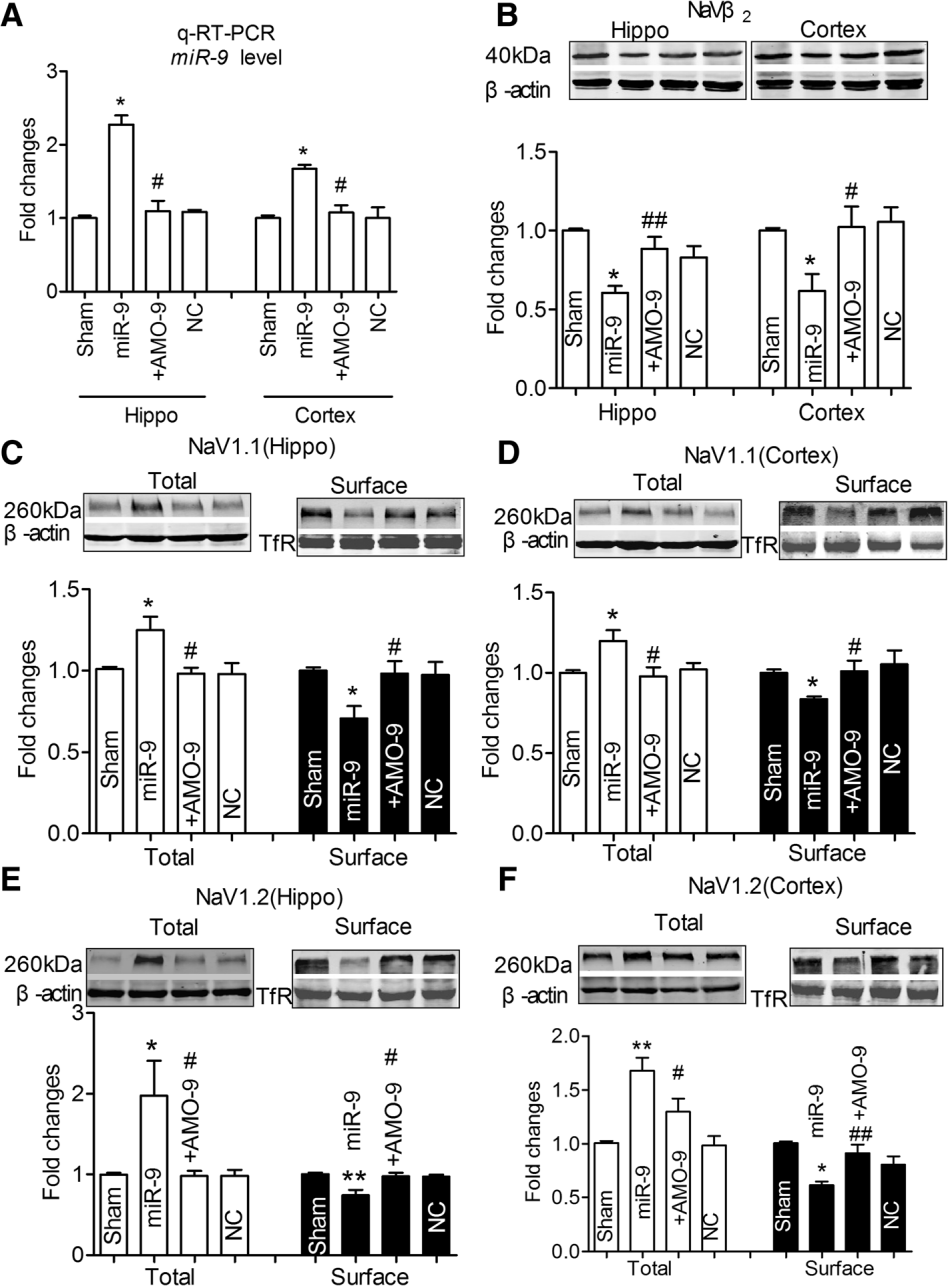
MiR-9 produces the disturbance of trafficking, cellular distribution of Nav1.1 and Nav1.2 in rats. **a**, Detection of miR-9 in hippocampi and cortices tissues after stereotaxic injection 8 weeks using qRT-PCR. Rats were transfected with lenti-pre- miR-9, lenti-pre-miR-9 + lenti-pre-AMO*-*miR-9, or NC. Data was shown by mean ± s.e.m from 6 rats for each group. *P < 0.05 *vs* NC; ^#^ *P* < 0.05 *vs* lenti-pre- miR-9. **b**, Navβ2 protein expressions in hippocampi and cortices tissues after treatment by lenti-pre-miR-9, lenti-pre-miR-9 + lenti-pre-AMO-miR-9, or NC for 8 weeks. **c**-**f**, Nav1.1 (C-D), Nav1.2 (E-F) total and surface protein expressions in hippocampi and cortices tissues after treatment by lenti-pre-miR-9, lenti-pre-miR-9 + lenti-pre-AMO*-*miR-9, or NC for 8 weeks. *P < 0.05,**P < 0.01 *vs* NC; ^#^ *P* < 0.05, ^##^ *P* < 0.01 *vs* lenti-pre- miR-9, mean ± s.e.m, *n* = 6

To verify the functional role of miR-9 on Nav1.1/Nav1.2 trafficking *in vivo*, miR-9 oligonucleotide carried by lentivirus vector (lenti-pre-miR-9) was injected directly into CA1 region of bilateral hippocampus of each rat and significantly higher expression of miR-9 in both hippocampi and cortices was observed at 8 weeks after injection compared with the negative control oligonucleotide (NC, Fig. 6a). Importantly, the expression of Navβ2 was also significantly decreased at meantime in lenti-pre-miR-9 group compared with control group, which was reversed by lenti-pre-AMO-miR-9 (Fig. 6b, *P* < 0.01). The surface expressions of both Nav1.1 and Nav1.2 proteins were markedly reduced in both hippocampi and cortices of rats with lenti-pre-miR-9 treatment, which was reversed by lenti-pre-AMO-miR-9 (Fig. 6c-f, *P* < 0.05), even though the total protein levels of Nav1.1 and Nav1.2 were increased in both hippocampi and cortices of rats with lenti-pre-miR-9 treatment, which were reversed by lenti-pre-AMO-miR-9 (Fig. 6c-f, *P* < 0.05). These data suggested that miR-9 plays an important role in trafficking and cellular distribution of Nav1.1/ Nav1.2.

### Reversal effect of AMO-miR-9 on trafficking defects of Nav1.1/Nav1.2 induced by 2VO

**Fig. 7:**
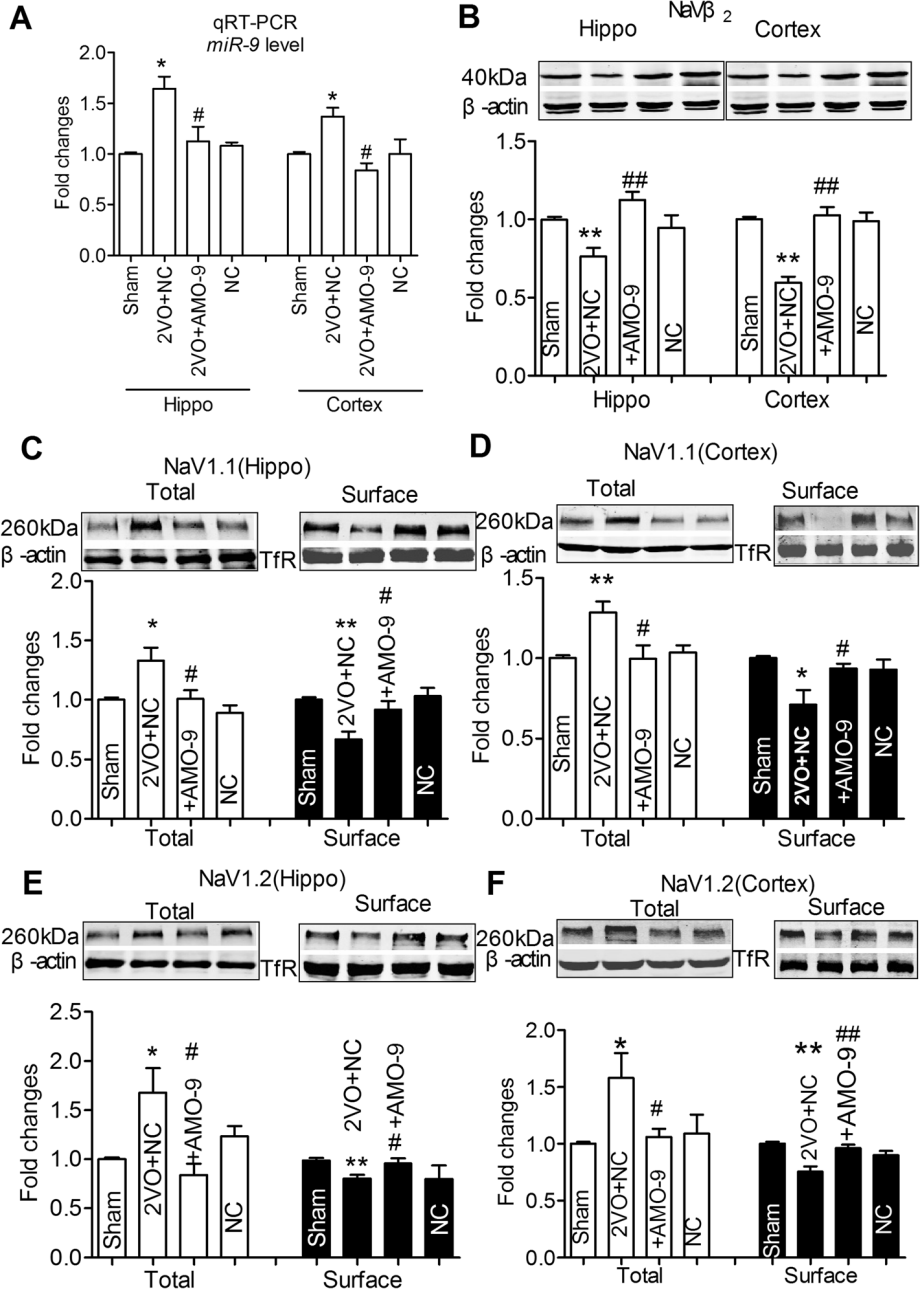
AMO-miR-9 prevented the disturbed trafficking of Nav1.1/Nav1.2 induced by 2VO. **a**, Detection of miR-9 in hippocampi and cortices tissues after stereotaxic injection 8 weeks using qRT-PCR. 2VO rats were transfected with lenti-pre-AMO*-*miR-9, or NC. Data was shown by mean ± s.e.m from 6 rats for each group. *P < 0.05*vs* sham; ^#^ *P* < 0.05 *vs* 2VO. **b**, Navβ2 protein expressions in hippocampi and cortices tissues in 2VO rats with or without lenti-pre-AMO*-*miR-9 treatment, or NC for 8 weeks. **c**-**f**, Nav1.1 (C-D), Nav1.2 (E-F) total and surface protein expressions in 2VO rats with or without lenti-pre-AMO*-*miR-9 treatment, or NC for 8 weeks. *P < 0.05,**P < 0.01 *vs* sham; ^#^ *P* < 0.05, ^##^ *P* < 0.01 *vs* 2VO, mean ± s.e.m, *n* = 6

Our data displayed that 2VO results in miR-9 increase, and over-expression of miR-9 then induces the trafficking defects of Nav1.1/Nav1.2 in vitro. In order to evaluate the protective effect of AMO-miR-9 on the onset of the trafficking defects of Nav1.1/Nav1.2 in 2VO rats, lenti-pre-AMO-miR-9 was injected into the hippocampus area. At 8 weeks after injection, miR-9 level was significantly decreased in both hippocampi and cortices compared with 2VO rats (Fig. 7a). To our interest, the decreased expression of Navβ2 was also reversed by lenti-pre-AMO-miR-9 treatment in the same brain regions (Fig. 7b, *P* < 0.01). And furthermore, the surface protein expression of Nav1.1 was increased and the total protein of Nav1.1 level was decreased by lenti-pre-AMO-miR-9 compared with that in 2VO control rats (Fig. 7c,d, *P* < 0.05). Similarly, lenti-pre-AMO-miR-9 effectively improved the impaired trafficking of Nav1.2 from cytoplasm to cell membrane under the same experimental condition (Fig. 7e and f, *P* < 0.05).

## Discussion

In this study, our observations have demonstrated, for the first time that CBH induces the trafficking defects of Nav1.1/Nav1.2 in hippocampi and cortices areas in 2VO rats, leading to the decrease in the expression of Navβ2 protein. Further study has shown that the increased miR-9 negatively regulated the expression of Navβ2 protein by binding to the target in CDS region of SCN2B gene. This observation provides a novel mechanism to modify the reduction in Nav1.1/Nav1.2 membrane trafficking, and careful monitoring the changes in miR-9 level and the expression for Nav1.1/Nav1.2 and targeted gene are considerably necessary during CBH.

### Molecular identity and trafficking characters of Nav1.1/Nav1.2 in rat brain after CBH

It has been known that Nav1.1, Nav1.2, and Nav1.6 are abundant in the central nervous system, whereas Nav1.3 is mostly present during embryonic stage [44]. Nav1.6 is concentrated in the axon initial segment (AIS) nodes of Ranvier and in proximal dendrites in many types of neurons [45]. Since cell surface levels of Nav1.1 and Nav1.2 subunits dramatically decrease in the brains of *BACE1*-trangenic mice although total Nav1.1 and Nav1.2 levels are elevated [12, 14], and that the axonal and surface levels of Nav1.2 are significantly increased in hippocampal neurons from *BACE1*-null mice [7]. In the present study, the expression of Nav1.1 and Nav1.2 of both surface and total protein were detected in hippocampi and cortices of rat following CBH and our data showed that CBH could induce trafficking defects of Nav1.1/Nav1.2 with significant increase in total protein levels of Nav1.1/Nav1.2 and the marked decrease in surface protein levels of Nav1.1/Nav1.2. The results are consistent with previous study performed using *BACE1*-trangenic mice [12].

Previous studies have demonstrated that Navβ2 subunit, an auxiliary subunit of Nav channel, participates in channel trafficking, re-localization, and interaction of both Nav1.1 [46, 47] and Nav1.2 [45]. Importantly, a recent study has also shown that the abnormal Navβ2 cleavage mediated by *BACE1* affects Nav1.1 and Nav1.2 surface trafficking differentially [48]. Furthermore, in AD status, the intracellular domain of Navβ2 functionally regulates the α-subunit of VGSCs and the elevated *BACE1* activity leading to decrease in surface levels of Nav1.1 in neuronal cells [49]. Interestingly, here we found that CBH not only impaired Nav1.1/Nav1.2 trafficking in rat hippocampi and cortices, but also downregulated the expression of Navβ2, suggesting Navβ2 may be more likely involved in the abnormal trafficking of both Nav1.1 and Nav1.2 induced by CBH. Of noted, previous studies have demonstrated that 2VO provokes chronic brain hypoxia and triggers spatial memory impairment in rats accompanied with elevation of *APP* and *BACE1* expression [24, 41]. Whether the impaired trafficking of Nav1.1/ Nav1.2 in the present study is associated with CBH induced high level of *APP* and *BACE1* is unknown and need to be elucidated further.

### Negative regulation of miR-9 on Navβ2 protein-mediated trafficking disturbance of Nav1.1/Nav1.2 in vitro

As far as we know, microRNAs are newly discovered and commonly considered as modulators of protein expression at post-transcriptional level, which are associated with the pathogenesis in multiple kinds of diseases [50–53]. In the present study, our observations have shown that the mRNA level for Navβ2 unaltered in hippocampi and cortices of 2VO rat but its targeted protein expression significantly decreased (Fig. 2b), indicating that the post-transcriptional regulation must be existed to modify Navβ2 expression. It has been documented in the literature that miR-9 is elevated in hippocampus [39] and temporal lobe cortex [54] of AD patients, whereas, the opposite observation is also presented in AD patients [55] from others,which was then demonstrated downregulation of miR-9 due to overexpression of Aβ in hippocampal cultures [56]. The discrepancies imply that the changes in miR-9 in AD depend presumably upon the variants inducers. In the present study, The major finding is that miR-9 is significantly up-regulated under CBH conditions in animal model (Fig. 2d).

Our study provides strong evidences that miR-9 increases in both hippocampi and cortices, and inhibited the expression of Navβ2, which in turn blocked the trafficking of Nav1.1 and Nav1.2 from cytoplasm to plasma membrane (Figs. 3, 4 and 5). However, why the total Nav1.1/Nav1.2 protein levels increase in cultured neuron following miR-9 treatment and how 2VO triggers Nav1.1/Nav1.2 total protein up-regulation remain unclear. The possible explanation may be due at least partially to a unknown mechanism increasing protein synthesis to compensate the trafficking defect-mediated the decrease in surface expression of Nav1.1/Nav1.2. But the detailed mechanism needs to be further studied.

In addition, our important finding here is that miR-9 regulates endogenous Navβ2 expression by targeting its coding sequence (CDS) region rather than not 3’UTR of SCN2B (Fig. 4). Additionally, another evidence collected from the current inverstigation demonstrate that the microRNA-mediated regulation is not limited to the 3’UTR, the functionality of target sites in the CDS also confirmed by previous studies [57–59], such as miR-24 [58], miR-296, miR-470, miR-134 [60], miR-126 [43], miR-181a [59], miR-148 [57] and miR-519 [61] that target sequences within the mRNA coding region have been reported to repress the biosynthesis of the encoded proteins in similar way. Our results provided another evidence that, microRNA-mediated regulation is not limited to target on the 3’UTR of genes, the functionality of target sites is also located in the CDS domain.

### Negative regulation of miR-9 on Navβ2 protein-mediated trafficking defects of Nav1.1/Nav1.2 *in vivo*

More importantly, our *in vivo* study supports the data collected from our *in vitro* observations that the upregulation of miR-9 induced by both CBH and lenti-pre-miR-9 could also disturb the trafficking of both Nav1.1/Nav1.2 by downregulation of Navβ2 expression. On the contrary, lenti-pre-AMO-miR-9 injection into hippocampus markedly prevents the abnormal trafficking of both Nav1.1 and Nav1.2 following either CBH or lenti-pre-miR-9 treated normal rats accompanied by increased Navβ2 expression. These results combination with our *in vitro* data suggested that the inhibition of miR-9 in hippocampi and cortices in CBH model rats would be a way to prevent sodium channel dysfunction after CBH. An understanding of miR-9*-*Navβ2-Nav1.1/Nav1.2 trafficking pathway could yield to the potential therapeutic targets for the prevention of abnormal electrical activation induced by CBH.

### Limitation and prospect

In the present study, though we have demonstrated the regulation effect of miR-9 on the trafficking of Nav1.1/Nav1.2 by inhibiting the expression of Navβ2 both *in vitro* and *in vivo,* we did not provide evidence whether these changes could induce abnormal sodium channel currents and its dynamics characteristics in hippocampi and cortices of 2VO rats. These need to be studied further.

## Conclusions

MiR-9 plays a key role in regulating the process of Nav1.1/Nav1.2 trafficking via targeting on Navβ2 protein in 2VO rats at post-transcriptional level, and inhibition of miR-9 may be a potentially-valuable approach to prevent the Nav1.1/Nav1.2 trafficking disturbance induced by CBH.

## Methods

### Animals

Male Sprague–Dawley rats (weight 220–260 g, obtained from the Animal Centre of the Second Affiliated Hospital of Harbin Medical University, Harbin, Heilongjiang Province, China) were housed at 23 ± 1 °C with 55 ± 5 % of humidity and maintained on 12 h dark–light artificial cycle (lights on at 07:00 A.M.) with food and water available *ad libium*. Rats used for operation of permanent, bilateral common carotid artery occlusion (2VO) and stereotaxic injection of the lentiviral vectors were anesthetized with chloral hydrate (300 mg/kg, intraperitoneal) and maintained by administrating 0.5-1.0 % isoflurane. The depth of anesthesia was monitored by detecting reflexes, heart rate and respiratory rate. Samples for qRT-PCR, and Western blot assay were obtained from the hippocampi and cortices of rats after anesthetized with chloral hydrate (500 mg/kg, intraperitoneal) following by confirmation of death by exsanguination. Tissues for primary neuron culturing were from neonatal SD rats after administration of 20 % isoflurane and confirmation of death by cervical dislocation. All animal procedures were approved by the Institutional Animal Care and Use Committee at Harbin Medical University (No.HMUIRB-2008-06) and the Institute of Laboratory Animal Science of China (A5655-01). All procedures were conformed to the Directive 2010/63/EU of the European Parliament.

### Permanent, bilateral common carotid artery occlusion (2VO) in the rat

The method for preparation of 2VO rat was according to the previous study [24, 62]. Briefly, after the rats were anaesthetized, the bilateral common carotid arteries of rats were exposed via a midline ventral incision, carefully separated from the vagal nerves, and permanently ligated with 5–0 silk suture. The wound was then closed and rats were allowed recovering from anesthesia before being returned to the animal facility.

### Primary hippocampal and cortical neuron culture

The hippocampi and cortices regions were removed from the postnatal day 0 (P0) rat pups. After tissues were dissected and triturated, they were plated onto cell plates precoated with 10 μg/mL poly-D-lysine (Sigma, St Louis, MO, USA) and cultured in the culture media containing neurobasal medium (Gibco, USA) with 2 % B27 supplement (Invitrogen, USA) and 10 % fetal bovine serum (FBS, HyClone, Logan, UT). After 3 days, the neurons were treated with 5 μM cytosine arabinoside (Sigma, St Louis, MO, USA) to inhibit astrocyte proliferation. For all experiments, the neurons were used at 14 days after plating [24].

### Synthesis of miR-9, AMO (anti-microRNA antisense oligodeoxyribonucleotide)-miR-9 and other various oligonucleotides

MiR-9 mimics (sense: 5’-UCUUUGGUUAUCUAGCUGUAUGA-3’; antisense: 5’-AUACAGCUAGAUAACCAAAGAUU-3’) and AMO-miR-9 (5’-UCAUACAGCUAGAUAACCAAAGA-3’) were synthesized by Shanghai GenePharma Co., Ltd (Shanghai, China). AMO-9 contains 2’-O-methyl modifications at every base and a 3’ C3-containing amino linker. Additionally, a scrambled RNA was used as a negative control (sense: 5’-UUCUCCGAACGUGUCACGUAA-3, and antisense: 5’-ACGUGACACGUUCGGAGAAUU-3’). The Navβ2-masking antisense oligodeoxynucleotides (ODNs) were synthesized by Shanghai Sangon Biological Engineering Technology and Service Co., Ltd. Navβ2 masking antisense-ODN-1 was 5’-ATGCCTTCGTCTTCTAGCTGC-3’, which masks the binding sites of miR-9, located in the position 336–358 of SCN2B CDS (coding sequence) region; Navβ2 masking antisense-ODN-2 was 5’TCCTCTTCGGTCTTCAGGTCA-3’ , which masks the binding sites of miR-9, located in the position of 575–597 of SCN2B CDS region. Five nucleotides or deoxynucleotides at both ends of the antisense molecules were locked by a methylene bridge connecting between the 2’-O- and the 4’-C atoms.

### Transfection procedures

Thirty pmol/mL miR-9 and/or AMO-9, ODNs or NC siRNAs were transfected into neonatal hippocampal and cortical neurons with X-treme GENE siRNA transfection reagent (Cat.#04476093001, Roche, USA) according to the manufacturer’s instructions. Forty-eight hours after transfection, cells were collected for total RNA isolation or protein purification.

### Construction of lentivirus vectors

Using the BLOCK-iT polII miR-RNAi expression vector with the EmGFP kit from invitrogen, three single-stranded DNA oligonucleotides were designed as follows: (1) pre-*miR-9* (“top strand” oligo: TGCTGTcTTTggTTaTcTagcTgTaTgaGTTTTGGCCACTGACTGACTcaTacagagaTaaccaaaga) and its complementary chain (“bottom strand” oligo: CCTGTcTTTggTTaTcTcTgTaTgaGTCAGTCAGTGGCCAAAACTcaTacagcTagaTaaccaaagaC); (2) pre-AMO-miR-9 (“top strand” oligo: TGCTGTcaTacagcTagaTaaccaaagaGTTTTGGCCACTGACTGACTcTTTggTTcTagcTgTaTga) and its complement (“bottom strand” oligo: CCTGTcaTacagcTagaaccaaagaGTCAGTCAGTGGCCAAAACTcTTTggTTaTcTagcTgTaTgaC); (3) negative control (“top strand” oligo: tgctgAAATGTACTGCGCGTGGAGACGTTTTGGCCACTGACTGACGTCTCCACGCAGTACATTT) and its complement (“bottom strand” oligo: cctgAAATGTACTGCGTGGAGACGTCAGTCAGTGGCCAAAACGTCTCCACGCGCAGTACATTTc). We then cloned the double-stranded oligonucleotides (ds oligo) generated by annealling the top and bottom strand oligos into the pcDNA™6.2-GW/± EmGFP-miR vector and transformed the ligated mixture into competent E. coli. After colony was purified and identified as the correct expression clone, the pre-microRNA expression cassette was transferred to other Gateway® adapted destination vectors utilizing PolII promoters and formed a new miRNA expression clone containing attR substrates. The vector was identified after analyzing the plasmid sequence (Invitrogen, USA). The titers of the vectors used for experiments were 9.25 × 10^8^ transducing U/ml. Virus suspensions were stored at −80 °C until use and were briefly centrifuged and kept on ice immediately before injection.

### Stereotaxic injection of the lentiviral vectors

After anaesthetized, rats were placed onto a stereotaxic frame (RWB Life Science Co. Ltd, China) described as previous study [24]. Injection coordinates relative to the bregma were as follows: AP (anteroposterior), −4.52 mm; ML (mediolateral), ±3.2 mm; DV (dorsoventral), −3.16 mm below the surface of dura using coordinates derived from the atlas of Paxinos and Watson. Two microliters (10,000 Tu/μl) lenti-pre-miR-9 and/or Lenti-pre-AMO-miR-9 were injected into CA1of the hippocampus using a 5 μl Hamilton syringe with a 33-gauge tip needle (Hamilton, Bonaduz, Switzerland). The needle was then maintained in the place for another 2 min after injection and then withdrawn very slowly to prevent the solution backflow.

### Dual luciferase reporter assay

Before the luciferase activity assay, plasmid design and construction was shown as in the below figure: a 707 bp fragment from the coding region of SCN2B containing the putative binding sequences for miR-9 (position 336–358 and position 575–597 of SCN2B CDS) was amplified by PCR, cloned into the pSICHECK-2-control vector.

Mutagenesis nucleotides were carried out using direct oligomer synthesis for the CDS region of Navβ2-binding site 1 and Navβ2-binding site 2. Point mutations were introduced into a possible miR-9 binding site located in the coding region of SCN2B (position 336–358 and position 575–597 of SCN2B CDS). MutSCN2B-1 represents that “GACGAAGG” was mutated “ATCGATCG” in the position 336–358 of SCN2B CDS, MutSCN2B-2 represents that “GACCGAAG” was mutated “ATCGATCG” in the position 575–597 of SCN2B CDS. MutSCN2B-1&2 represents that both sites were mutated. All constructs were sequence verified. Rat SCN2B CDS and mutSCN2B CDS sequences were shown as following: SCN2B CDS sequences (bold nucleotide showed the putative binding sequences for miR-9): ATGCACAGGGATGCCTGGCTACCTCGCCCTGCCTTCAGCCTCACGGGGCTCAGTCTGTTTTTCTCTTTGGTGCCCTCGGGGCGGAGCATGGAAGTCACAGTCCCCACCACTCTTAGTGTCCTCAACGGGTCTGATACCCGCCTGCCCTGTACCTTCAACTCCTGCTATACCGTGAACCACAAGCAGTTCTCTCTGAACTGGACTTACCAGGAGTGTAGCAATTGCTCAGAGGAGATGTTCCTCCAGTTCCGAATGAAGATCATCAACCTGAAGCTGGAGCGGTTTGGAGACCGCGTAGAGTTCTCGGGGAACCCCAGTAGTACGACGTGTCAGTGACTCTAAAGAA(CGTGCAGCTAGAA**GACGAAGG**C)ATTTACAACTGCTAATCACCAACCCTCCAGACCGCCACCGTGGCCATGGCAAGATCTACCTGCAGGTCCTTCTAGAAGGCCCCCAGAGCGGGACTCCACGGTGGCAGTCATCGTGGGTGCCTCAGTGGGGGGTTTCCTGGCTGTGGTCATCTTGGTGCTGATGGTGGTCAAATGTGTGAGGAGGAAAAAAGAGCAGAAGCTGAGC(ACGGATGACCTGAA**GACCGAAG**A)GGAAGGCAAGACGGATGGCGAGGGCAACGCGGAAGATGGCGCCAAGTAACCGGAAGCTTGCCCTGAAGCCCCTTCCTGTGTCCTGTCTCCTCTCACTCTCTGCCCTGT; mutSCN2B CDS (bold nucleotide): ATGCACAGGGATGCCTGGCTACCTCGCCCTGCCTTCAGCCTCACGGGGCTCAGTCTGTTTTTCTCTTTGGTGCCCTCGGGGCGGAGCATGGAAGTCACAGTCCCCACCACTCTTAGTGTCCTCAACGGGTCTGATACCCGCCTGCCCTGTACCTTCAACTCCTGCTATACCGTGAACCACAAGCAGTTCTCTCTGAACTGGACTTACCAGGAGTGTAGCAATTGCTCAGAGGAGATGTTCCTCCAGTTCCGAATGAAGATCATCAACCTGAAGCTGGAGCGGTTTGGAGACCGCGTAGAGTTCTCGGGGAACCCCAGTAAGTACGACGTGTCAGTGACTCTAAAGAA(CGTGCAGCTAGAA**ATCGATCG**C)ATTTACAACTGCTACATCACCAACCCTCCAGACCGCCACCGTGGCCATGGCAAGATCTACCTGCAGGTCCTTCTAGAAGTGCCCCCAGAGCGGGACTCCACGGTGGCAGTCATCGTGGGTGCCTCAGTGGGGGGTTTCCTGGCTGTGGTCATCTTGGTGCTGATGGTGGTCAAATGTGTGAGGAGGAAAAAAGAGCAGAAGCTGAGC(ACGGATGACCTGAA**ATCGATCG**A)GGAAGGCAAGACGGATGGCGAGGGCAACGCGGAAGATGGCGCCAAGTAACCGGAAGCTTGCCCTGAAGCCCCTTCCTGTGTCCTGTCTCCTCTCACTCTCTGCCCTGT

The sequence of miR-9 mimic is 5’-UCUUUGGUUAUCUAGCUGUAUGA-3’ (synthesized based on the sequence of rno miR-9 (miRBase Accession No. MIMAT0000781)); that of miR-NC is 5’-UUCUCCGAACGUGUCACGUAA-3’; the sequence of the antisense 2’-O-methyl (2’-O-Me) oligonucleotide for miR-9 is 5’-UCAUACAGCUAGAUAACCAAAGA-3’, that of inhibitor-NC is 5’UUCUCCGAACGUGUCACGUTT-3’; HEK293T cells (plated at 40 % ~ 50 % confluence) were transfected with 20 μmol/l miR*-*9, AMO-miR*-*9, or negative control siRNAs (NC) as well as 0.5 μg of psi-CHECK^TM^-2-target DNA (firefly luciferase vector) and 1 μl blank plasmid using lipofectamine 2000 (Invitrogen,USA) transfection reagent according to the manufacturer’s instructions. After 48 h of transfection, Firefly and renilla luciferase activities, as indicated by relative luminescence units (RLU) were determined using luciferase assay kits (Cat.#E1910, Promega, USA) and luminometer (GloMax^TM^ 20/20, Promega, USA) according to the manufacturer's instructions.

### Quantitative real-time PCR- (qRT-PCR)

Total RNA was purified with the Trizol Reagent (Invitrogen, USA), according to the manufacturer’s instructions as described previously [24]. *MiR-9* level was quantified by the TaqMan® MicroRNA Reverse Transcription Kit (Cat.# 000583, ABI, Roche, Branchburg, NJ) and the TaqMan® Gene Expression Master Mix (Cat.# 1108123, Applied Biosystems). The TaqMan qRT–PCR probes and primers for *miR-9*, were designed by Applied Biosystems [63]. U6 was used as an internal control. The SYBR Green PCR Master Mix Kit (Applied Biosystems, Cat#4309155) was used for real-time PCR to quantify the SCN2B mRNA in our study. β-actin was used as an internal control. Primers are as following: *SCN2B* forward: CTCTCTGAACTGGACTTACC and *SCN2B* reverse: GGTTGGTGATGTAGCAGTTG; β-actin forward: GGAAATCGTGCGTGACATTA and β-actin reverse: AGGAAGGAAGGCTGGAAGAG. All reactions were performed in triplicate, and the expressions of microRNAs data were shown as Delta-Delta Ct method.

### Western blot

Both the total protein and surface protein samples were extracted from hippocampi and cortices of rats or primary cultured neurons for immunoblotting analysis. For the total protein analysis, frozen tissue was homogenized with 1000 μl solution contained 40 % SDS, 60 % RIPA and 1 % protease inhibitor in each 200 mg brain tissue. The homogenate was then centrifuged at 13,500 rpm for 30 min and the supernatants (containing cytosolic and membrane fractions) were collected. The method of surface protein extraction was using Mem-PER Eukaryotic Membrane Protein Extraction Reagent Kit (Cat# 89826, Pierce Biotechnology, USA) according to the manufacturer's instructions. Protein concentrations were measured spectrophotometrically using a BCA kit (Universal Microplate Spectrophotometer; Bio-Tek Instruments, Winooski, VT, USA). Protein samples were fractionated by SDS-PAGE (10 % polyacrylamide gels for sodium channels) then transferred to PVDF membrane. The primary anti-Nav1.1, Nav1.2, Navβ2 antibodies (Cat# ASC-001,1:200, RRID:AB_2040003; Cat# ASC-002,1:200, RRID:AB_2040005; Cat# ASC-007, 1:200, RRID:AB_2040011, Alomone Labs, Jerusalem, Israel) were used and β-actin (Kangcheng, Shanghai, China) β-actin (Kangcheng, Shanghai, China) was selected as an internal control of total proteins, mouse anti-human transferrin receptor (TfR) (Cat# 13–6800, RRID:AB_2040011, Invitrogen, USA) was selected as an internal control of surface proteins. Western blot bands were captured on the Odyssey Infrared Imaging System (LI-COR Biosciences, Lincoln, NE, USA) and quantified with Odyssey v1.2 software by measuring the band intensity (area × OD) in each group and normalizing to the internal control.

### Immunocytochemistry staining

The cultured neonatal rat neurons were transcardially perfused by 4 % buffered paraformaldehyde, pH7.4. After blocking, cultured neonatal rat neurons were incubated with the anti-β-Tubulin III (neuronal) antibody (Cat no. T8578; 1:5000; Sigma, Saint Louis, USA) or anti- Nav1.1, Nav1.2, Navβ2 antibodies (Alomone Labs, Jerusalem, Israel) overnight at 4 °C, and then the cultured neonatal rat neurons were washed and incubated with the secondary antibodies conjugated to Alexa Fluor 488 and Alexa Fluor 594 (Molecular Probes, Eugene, OR, USA) for 1 h at room temperature.

### Statistical analysis

Data were described as mean ± s.e.m for experimental data. The two-tailed Student’s *t*-test was applied for comparisons between the two groups. Multi-group’s comparisions were performed by One-way ANOVA. SPSS19.0 software was used for all statistical analyses. *P* < 0.05 was considered significant.

## Abbreviations

VGSC: Voltage-gated sodium channel
AD: Alzheimer's disease
CBH: Chronic brain hypoperfusion
CNS: Central nerve system
2VO: Bilateral common carotid artery occlusion
ODNs: Oligodeoxynucleotides
AMO: Anti-microRNA antisense oligodeoxyribonucleotide
CDS: Coding sequence
TfR: Transferrin receptor
AIS: Axon initial segment
q-RT-PCR: Quantitative real-time PCR
